# Garlic Potyviruses Are Translocated to the True Seeds through the Vegetative and Reproductive Systems of the Mother Plant

**DOI:** 10.3390/v14102092

**Published:** 2022-09-21

**Authors:** Einat Shemesh-Mayer, Dana Gelbart, Eduard Belausov, Nisan Sher, Ahuva Daus, Haim D. Rabinowitch, Rina Kamenetsky-Goldstein

**Affiliations:** 1 Institute of Plant Sciences, Agricultural Research Organization—The Volcani Institute, Risho LeZion 7505101, Israel; 2 Robert H. Smith Faculty of Agricultural, Food, and Environmental Quality Sciences, The Hebrew University of Jerusalem, Jerusalem 9190501, Israel

**Keywords:** *Allium sativum*, virus transmission, fluorescence in situ hybridization (FISH)

## Abstract

Garlic lost its ability to produce true seeds millennia ago, and today non-fertile commercial cultivars are propagated only vegetatively. Garlic viruses are commonly carried over from one generation of vegetative propagules to the other, while nematodes and arthropods further transmit the pathogens from infected to healthy plants. A recent breakthrough in the production of true (botanical) garlic seeds resulted in rapid scientific progress, but the question of whether viruses are transmitted via seeds remains open and is important for the further development of commercial seed production. We combined morpho-physiological analysis, fluorescence in situ hybridization (FISH), and PCR analysis to follow potyvirus localization and translocation within garlic fertile plants and seeds. Spatial distribution was recorded in both vegetative and reproductive organs. We conclude that garlic potyviruses are translocated to the seeds from the infected mother plant during flower development and post-fertilization, while pollen remains virus-free and does not contribute to seed infection. Therefore, the main practical goal for virus-clean seed production in garlic is the careful maintenance of virus-free mother plants. Although garlic pollen is free of potyviral infection, the male parents’ plants also need to be protected from contamination, since viral infection weakens plants, reducing flowering ability and pollen production.

## 1. Introduction

Garlic, *Allium sativum* L., is one of the oldest vegetables in the human diet. Originating in East–Central Asia, this important crop and nutraceutical condiment is currently farmed worldwide, in many climatic zones, for its bulbs, green leaves, and young floral stalks [1]. China, the world’s largest garlic producer [2], provides the largest share of the global 2.5 M tons of international trade, totaling US $3 billion in 2021. Garlic lost its blooming potential and sexual fertility already millennia ago [3] due to ongoing selection for larger cloves and bulbs, with the consequent diversion of most nutritional and energy resources to the vegetative storage organs. Thus, only non-fertile commercial cultivars are known, and vegetative propagation is exclusively practiced worldwide [4].

Similar to other vegetatively propagated crops, most commercial garlic clones harbor numerous viruses, thus suffering 25 to 50% yield losses and reduced quality. The viral infection is further promoted by international trade and germplasm exchange [5,6]. Similar to other vegetatively propagated crops (e.g., potatoes) [7], garlic viruses are commonly carried over from one generation of vegetative propagules to the other, while nematodes and arthropods further transmit the pathogens from infected to healthy plants [8] within the production field, from adjacent garlic fields, and/or from wild and cultivated flora to the garlic production fields.

Garlic plants are commonly infected by the members of three virus families: Potyviridae (genus Potyvirus), Betaflexiviridae (genus Carlavirus), and Alphaflexiviridae (genus Allexivirus) [8]. The most harmful aphids-transmitted potyviruses, onion yellow dwarf virus (OYDV) and the leek yellow-strip virus (LYSV), infest most garlic fields. In many parts of the world, garlic common latent virus (GCLV) and shallot latent virus (SLV), the most commonly spread Carlaviruses, are transmitted by garlic mites. Infection with one or more of the eight Allexivirus species, namely garlic mite-borne filamentous virus (GarMbFV), garlic virus A (GarV-A), garlic virus B (GarV-B), garlic virus C (GarV-C), garlic virus D (GarV-D), garlic virus E (GarV-E), garlic virus X (GarV-X), and shallot virus X (ShVX), cause only some non-severe yield losses [9]. Lately, new garlic-infecting viruses in garlic were identified, e.g., asparagus virus 3 (AV3) in Australia [6], garlic yellow mosaic-associated virus (GYMaV), in Brazil [10], and cucumber mosaic virus (CMV) in Korea [11].

In the absence of seeds, only vegetative cloves and aerial bulbils (topsets) are used for garlic propagation. This vegetative propagation, however, suffers many disadvantages, such as a low multiplication rate and voluminous storage space. In storage, heavy losses may occur due to deterioration and loss of viability, sprouting, and pathogens infection. Moreover, vegetative propagation leads to the perpetuation of diseases, severe sanitary issues, and heavy losses in yield and quality [12,13].

In an attempt to reduce infection load, garlic propagation by aerial bulbils and biotechnological methods are becoming popular [14,15,16]. Some reports suggest that bulbils are less infected by viruses than cloves and, therefore, possess the best potential for the propagation of healthy bulbs [17,18]. However, others reported that bulbils are actually heavily infested [19,20], thus adding no extra benefit to garlic production. Our recent research provides clear evidence of the presence of harmful potyviruses in garlic inflorescence meristem and reaffirms that fully mature aerial bulbils are heavily infested with viruses [21].

In the last decades, the breakthrough in fertility restoration and production of true garlic seeds resulted in rapid scientific progress on one hand and opened the way for advanced research, as well as breeding of modern and improved garlic varieties on the other [14]. The question of whether garlic-infecting viruses are transmitted via true seeds, however, remains open and is important for the further development of commercial seed production and garlic farming. The restoration of garlic fertility provided a unique opportunity to investigate virus presence in garlic floral reproductive tissues and seeds and compare seedlings’ infestation in seed- and clonal-propagated plants.

In general, seed-borne diseases and the quality of seeds have a significant impact on the development and trade of crops [22]. About 15% of known plant viruses are transmitted through seed, thus serving as an initial source of inoculum for the secondary spread within an area and neighboring and faraway lands through vectors [23,24]. Seed-borne viruses are transmitted either as surface contaminants; for example, the tobacco mosaic virus TMV contaminates the tomato seed testa [25], while most seed-borne viruses are carried in the embryo, endosperm, or seed coat [23]. In most cases, viruses do not integrate into the host genome and often do not pass efficiently through the germline, which serves as an effective barrier that blocks the transmission of most viruses from one generation to the next generation [23].

The ability of viruses to infect male and female gametophytes is crucial in determining the virus transmission potential through the embryo [26]. Additionally, temperature, genotypic makeup, and the time of infection are critical in determining whether seed infection is successful or not. Early virus contamination allows for sufficient time to complete the infection of the embryonic tissue before its separation from the mother plant. However, the rate of contamination varies from plant to plant, from season to season, and from field to field [27,28].

In the *Allium* species, OYDV is considered the most economically devastating pathogen [29,30]. Although OYDV was detected in the pollen of infected onion plants [31], its transmission through seed has not been detected [32,33]. Moreover, iris yellow spot virus (IYSV) is not transmitted by onion true seed [34,35].

If garlic true seeds transmit viruses, it is important to clarify their localization and what the sources of contamination are: is it the mother’s reproductive systems, the pollen, or both? In the case of surface localization, certain disinfection procedures can readily inactivate such external virus associations and their translocation to the germinating seedlings. However, where viruses infect inner tissues (e.g., cucumber and melon) [36], the eradication of seed-borne viruses is significantly more challenging.

We hereby report on the presence of potyviruses both in the vegetative and reproductive plant tissues and in true garlic seeds. This is the first report on virus transmission in sexually propagated garlic.

## 2. Materials and Methods

### 2.1. Plant Material and Sampling

**Figure 1:**
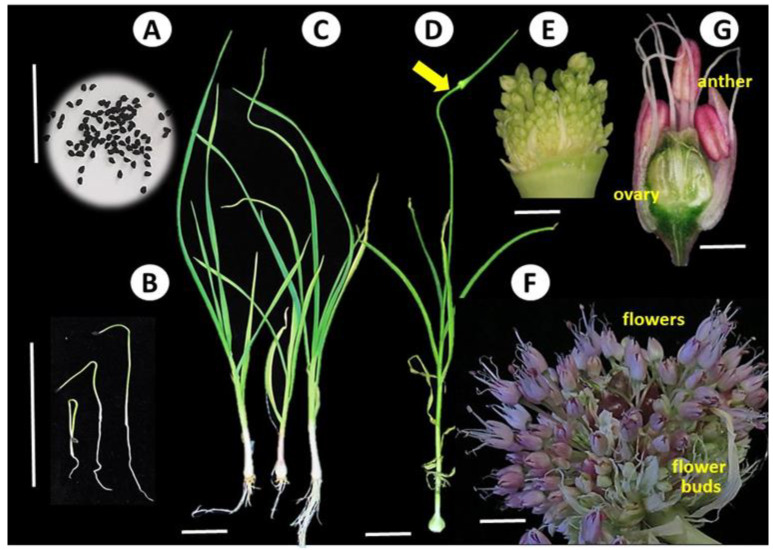
Representative samples of fertile garlic genotypes from true seed to flowering at different stages of development. Scale: A = 3 cm, B, D = 5 cm, C = 2 cm; E, F, G = 1 cm. (**A**) true garlic seeds. (**B**) five days old seedlings. (**C**) adult plants from cloves. (**D**) pre-anthesis garlic plant, with scape and spathe (arrow) visible. (**E**) flowers at early stages of development in the young inflorescence. (**F**) matured inflorescence, older flowers with anthers are visible at the umbel top, young flowers and flower buds are located in the lower part of the inflorescence. (**G**) cross-section of a post-anthesis flower. Anthers and an ovary are visible.

True garlic seeds harvested from various garlic accessions are kept at the Israeli Gene Bank. Selfed seed populations of the fertile accession #87 from the seasons of 2015–2017 (Figure 1) were sown in a growing mixture consisting of 50% peat, 30% ground coconut shell: 20% Styrofoam (Tuff Merom Golan, Israel) in 40 L plastic containers, with 200 seeds per container. The plants were grown in a 30% shaded net house.

A collection of fertile garlic clones is maintained in ARO, The Volcani Center, Rishon Lezion, Israel. In July 2021, freshly harvested bulbs of 12 of the fertile seed-producing accessions were harvested and stored under ambient conditions in a roofed open shed. Cloves were planted in November 2021 at 80 plants/m^2^ in 200 L containers filled with a mixture of 50% ground coconut shell: 20% volcanic tuff particles: 20 peat: 10% compost (Even Ari Green, Israel).

In February 2022, the plants of 12 fertile accessions were screened for the presence of potyviruses. Sections of the fourth youngest leaf were collected from two plants of each accession, and both OYDV and LYSV were detected by using PCR. Two accessions, 3376 and 3379, were confirmed as infected by potyviruses and used for future analyses. Samples from three plants of these lines were collected at the stages of bulbil sprouting, mature leaves, young inflorescences, and individual young and mature flowers and seeds. In addition, at least 20 flowers, seeds, or seedling were pooled for the RNA extraction (Figure 1).

### 2.2. RNA Fluorescence In Situ Hybridization

Single-stranded DNA oligos of 24 nucleotides labeled with the fluorophore cyanine-Cy3 were used as a probe for RNA hybridization. The labeled probe (5′-TGC TGT GTG CCT CTC CGT GTC CTC-3′) was designed as a specific universal primer RS1 for the detection of conserved potyviruses’ genomic sequences, including LYSV and OYDV.

Our initial attempts to perform virus analysis using published fluorescence in situ hybridization (FISH) protocols [37,38] failed. Therefore, a series of calibration experiments for garlic tissues, using probe concentrations of 3 or 5 pmol/mL and hybridization temperatures of 25, 37, or 50 °C, were carried out.

All tissue samples were fixed overnight in Carnoy’s fixative (6:3:1 mixture of chloroform: ethanol: glacial acetic acid), followed by two decolorization steps in 6% H_2_O_2_ solution in EtOH for 1 h. Then the sampled tissues were pre-hybridized in hybridization buffer (20 mM Tris–HCl, pH 8.0, 0.9 M NaCl, 0.01% sodium dodecyl sulfate (SDS), 30% formamide) for 1 h, followed by overnight hybridization in an incubator equipped with an orbital shaker (25 rpm) at 25, 37, or 50 °C in a hybridization buffer containing the fluorescent probe (3 or 5 pmol/mL).

The hybridized samples were washed twice in sodium chlorides/sodium citrate (SSC) mixed with 0.1% SDS solution for 10 min, and once with a mixture of 0.5 × SSC and 0.1% SDS for 10 min at room temperature. Samples were stored in SSC at 4 °C, until studied under a Leica SP8 laser scanning microscope (Leica, Wetzlar, Germany) that was equipped with solid-state lasers with 405 and 552 nm light, HCX PL APO CS 10 × /0.40 or HC PL APO CS2 20 × /0.75 objective (Leica, Wetzlar, Germany), and Leica Application Suite X software (LASX, Leica, Wetzlar, Germany). Then 4′,6-Diamidino-2-phenylindole (DAPI) and Cy3 emission signals were detected with PMT and HyD (hybrid) detectors in ranges of 415–490 and 560–660 nm, respectively.

Control tissues were similarly treated, but the fluorescent probe was replaced by water.

### 2.3. 4′,6-Diamidino-2-phenylindole (DAPI) Staining

DAPI staining that indicates membrane viability is used extensively in fluorescence microscopy to stain both live and fixed cells. In this study, staining with 0.1 mM DAPI solution (Abcam plc., Zotal Ltd., HaBarzel St 4, Tel Aviv-Yafo, Israel) was employed following RNA hybridization with Cy3 (see above) and examined under a confocal microscope.

### 2.4. Histology of the Reproductive Organs

For plastic embedding, flowers fixed in FAA were dehydrated in a graded series of ethanol concentrations (25, 50, 75, 90, and 100%), followed by immersion in acetone and gradual replacement by LR-White resin (Sigma-Aldrich, St-Louis, MO, USA). Following polymerization at 60 °C for 48–72 h, 2 µm–thick slices were obtained by using a rotary microtome (LeicaRM2245) and stained with 0.05% toluidine blue.

For paraffin embedding, samples were fixed in FAA, dehydrated in alcohol series as described above, cleared with xylene, and embedded in paraffin, using Paraplast Plus tissue-embedding medium. Xylene was gradually replaced with paraffin (melting point of 58–60 °C) at 60 °C for 4 four days. Cross-sections of 12 μm–thick slices were cut, using a Leica RM2245 microtome (Leica Bio-systems, Wetzlar, Germany), and stained with Safranin-Fast Green. The tissue slices were studied under a light microscope (Leica DMLB, Wetzlar, Germany).

### 2.5. Viral RNA Extraction, cDNA Synthesis, and PCR Analysis

Viral RNA was extracted from 100 mg of fresh tissues, using the AccuPreo Viral RNA Extraction Kit (Bioneer Corporation, Daejeon, South Korea), according to the manufacturer’s instructions. The RNA concentrations were determined spectrophotometrically (Nanodrop ND-1000, NanoDrop Technologies Wilmington, USA). The remaining RNAs served as templates for cDNA synthesis, using 1 µg total RNA, 0.1 µL random primer, and 0.9 µL oligo dT primer (RevertaidTMfirst-strand, Fermentas, ON, Canada). The PCR reactions were performed in 20 μL, containing 1 μL of cDNA as template, 10 pmol of each primer, 0.2 mM of each dNTP, 2 mM MgCl2, 0.5 U of Taq DNA polymerase, and 1× PCR buffer, using a T-personal thermal cycler (Biometra, Analytik Jena, Gottingen, Germany). PCRs consisted of an initial incubation at 94 °C for 3 min, followed by 35 cycles of denaturation at 94 °C for 30 s, annealing at 60 °C for 30 s, and 72 °C for 1 min. Final elongation at 72 °C was carried out for 10 min. PCR amplification products were visualized by electrophoresis in 1.0% agarose gels stained with ethidium bromide. The specific primers for LYSV and OYDV were previously reported [21], while the primers for Allexiviruses and Carlaviruses were designed and synthesized for this study (Table 1). Species of Allexiviruses and species of Carlaviruses were detected combined due to their similarity.

Target amplification was confirmed by cloning and Sanger sequencing (Hylabs, Israel) of the PCR reaction products.

## 3. Results

### 3.1. Calibration of FISH Technique for Virus Detection in Garlic

**Figure 2:**
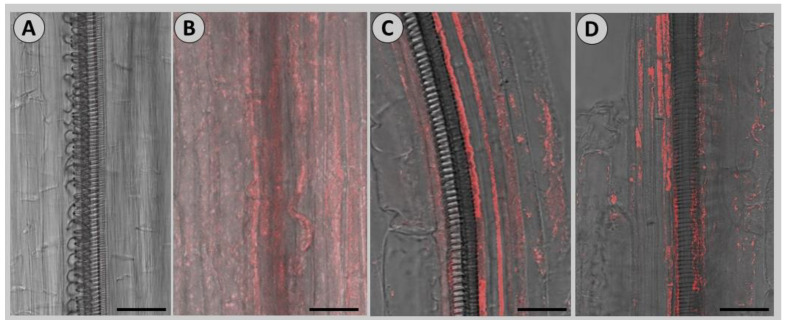
Calibration of FISH protocol for garlic leaf tissues. Scale = 80 µm. (**A**) Control, no probe. (**B**) Probe concentration 3 pmol/mL, overnight hybridization at 25 or 37 °C. (**C**) Probe concentration 3 pmol/mL, overnight hybridization at 50 °C. (**D**) Probe concentration 5 pmol/mL, overnight hybridization at 50 °C.

To optimize the FISH protocol for garlic tissues, we hybridized Cy-3 probe at 3 or 5 pmol/mL overnight at 25, 37, or 50 °C. The fluorescent signals were highly visible when 3 pmol/mL Cy-3 was hybridized at 50 °C (Figure 2). Hence, we followed this protocol throughout.

### 3.2. Identification of Viral Infection by PCR Analysis

In January 2022, we screened twelve selected garlic fertile clones and three seed populations for the presence of potyviruses. Plants of two vegetatively propagated accessions, 3376 and 3379, were identified as infected.

In the next PCR analysis, we tested specific tissues (Table 1) of those selected genotypes and seeds from three harvests (2015–2017) for the presence of LYSV and OYDV Potyviruses, Carlaviruses, and Allexiviruses. Our findings show that all assayed tissues were infected with *Allium*-specific Allexiviruses, which are considered harmless to the crops despite the induced mosaic and ringspot symptoms [39,40]. Allexiviruses’ RNA is closely associated with garlic plant transcriptome [41], thus suggesting their potential ability to incorporate into the host genome. Additional research is required for our understanding of co-evolution of this group with garlic and other *Allium* species and the transmission of Allexiviruses in vegetative and reproductive organs.

Carlaviruses were detected only in one young inflorescence and in one seed population, while the most harmful and dangerous potyviruses, LYSV and OYDV, were detected in all vegetative and reproductive organs of the selected clonally propagated genotypes 3376 and 3379. Seeds and seedlings also contained potyviruses. Interestingly, seeds from the 2016 harvest did not contain potyviruses, but their presence was detected in two other seed populations (Table 2).

The results of PCR analysis of LYSDV, OYDV, GarV-D, and GCLV were cross-validated with Sanger sequencing.

### 3.3. Anatomy of Reproductive Organs

**Figure 3:**
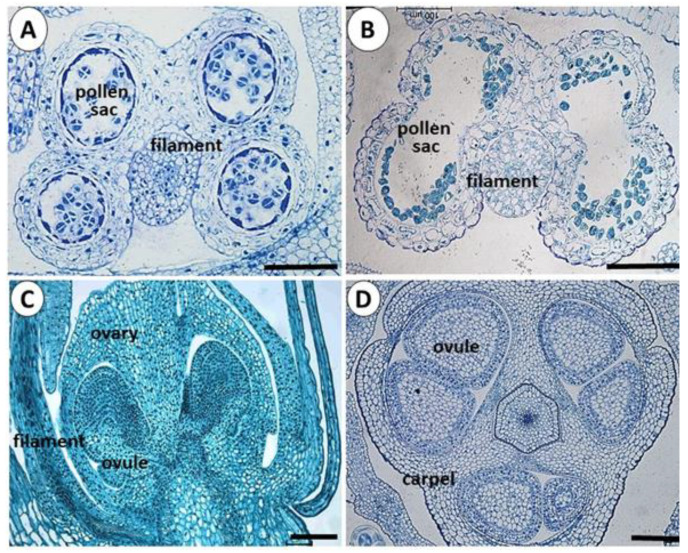
Structure of the garlic male and female organs. Scale = 100 μm. (**A**) Cross-section of anther during pollen meiosis. (**B**) Cross-section of anther after pollen maturation. (**C**) Longitudinal section of gynoecium during ovule development. Stamen filament is attached to the ovary basis. (**D**) Cross-section of the developing ovary.

Individual garlic flowers comprise perianth, androecium, and gynoecium. The androecium consists of six stamens composed of a filament and a bi-lobed anther; each lobe contains two pollen sacs (Figure 3A). When the pollen sacs are ripe, the anther releases the matured pollen (Figure 3B).

The gynoecium develops in the center of the flower and consists of ovary and style. The ovary includes the carpel, divided into three locules, containing two ovules in each locule (Figure 3C,D).

### 3.4. FISH Identification of Potyviruses in Vegetative and Reproductive Tissues

**Figure 4:**
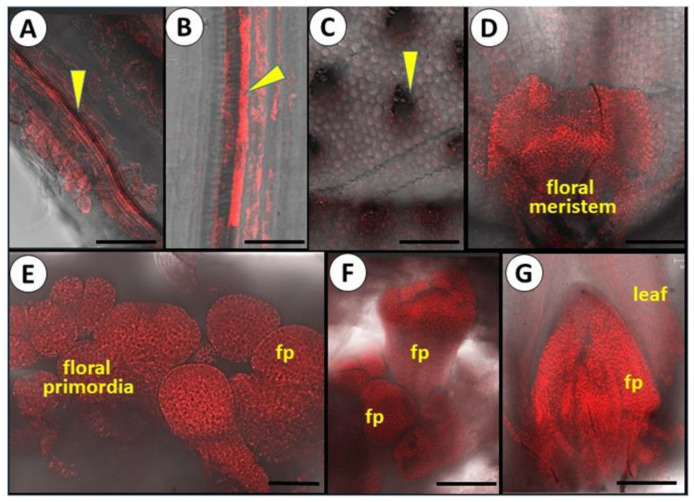
Microphotographs of FISH analysis of potyviruses spatial distribution in vegetative and reproductive tissues of fertile garlic accession. February/March 2022. Scale: A, B = 35 µm; C, F, G = 200 µm; D = 250 µm; E = 150 µm. (**A**) First seedling true leaf. Both phloem (arrow) and parenchyma are infected. (**B**) Young foliage leaf originated from the bulb clove. The phloem vascular tissue (arrow) is infected by potyviruses. (**C**) Cross-section of floral scape. The phloem vascular bundles (arrow) are infected. (**D**) Floral meristem, initiation stage, infected by potyviruses. (**E**) Differentiation of floral primordia (fp) infected by potyviruses. (**F**) Infected differentiating flower primordia; (**G**) Infected differentiated young flower.

The FISH analyses during the vegetative and reproductive development of garlic revealed potyviruses in foliage leaves, floral stem, floral meristem, and developing flowers (Figure 4). In seedlings, mature leaves, and floral stems, viruses were found mainly in vascular bundles (Figure 4A–C), while after the transition to reproductive development, strong fluorescent signals were observed in the apical meristem and young flower primordia (Figure 4D–G), where the vascular system is not developed yet. Interestingly, in young seedlings, both vascular and parenchymatic foliar tissues were infected, while mature foliage leaves showed strong signals mainly in the phloem. We thus conclude that, much like other plants [42], in garlic, potyviral transport occurs either in phloem tissues or from cell-to-cell movement. Young cells with thin cell walls can be readily infected via plasmodesmata, thus leading to systemic infection [43]. Intercellular channels—plasmadesmata—enable the trafficking of the metabolites and are also utilized by viruses for the systemic infection of hosts [44]. In the garlic reproductive tissues, this trafficking results in virus transport to the young developing flowers.

**Figure 5:**
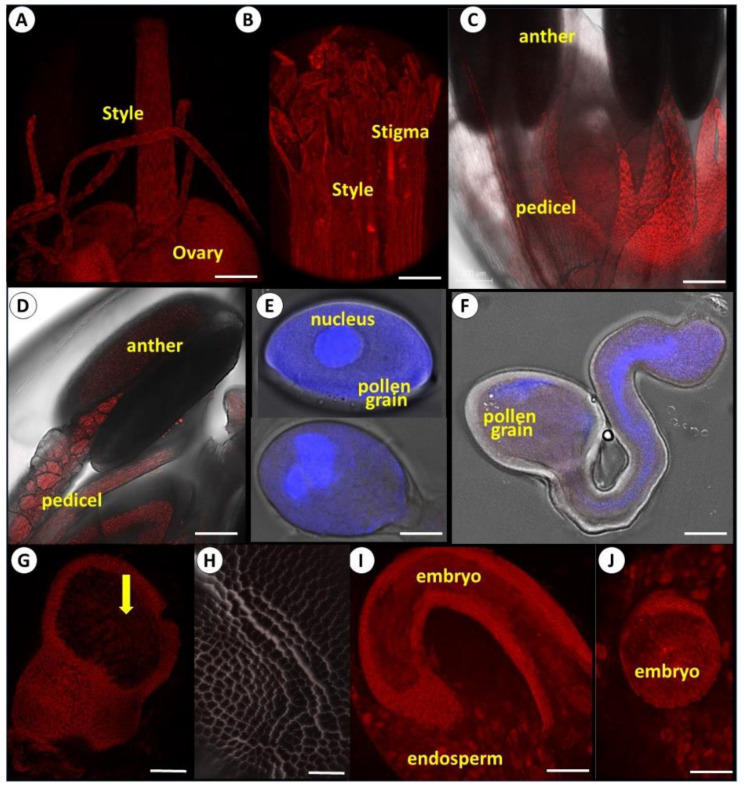
Representative confocal microscopy images of FISH tracing potyviruses’ spatial distribution in reproductive tissues and seeds of fertile garlic. Virus RNA was labeled by Cy-3. Pollen in E and F are stained with DAPI in addition to Cy-3. Scale: A, C, D, G, H, I = 250 µm; B = 50 µm; E, F = 10 µm; J = 150 µm. (**A**) Female flower organs, ovaries, style, and stigma. (**B**) Close-up of style, stigma, and papilla. A strong signal was recorded. (**C**) Male organs of garlic flower. The filaments are infected, while no signal is visible in the anthers. (**D**) Close-up shot of the anther. (**E**) Pollen grains before and after germination; vegetative and generative nucleus cells are colored by DAPI, and no signal from potyviruses is recorded. (**F**) Pollen tubes. Vegetative and generative nuclei cells are colored by DAPI. No signal from potyviruses is recorded. (**G**) Seed developing inside an ovary. (**H**) Seed coat, no visible signal. (**I**) Longitudinal section of viable seed before germination. Both embryo and endosperm are infected with potyviruses. (**J**) Cross-section of seed.

With flower development and maturation, the signals of viral RNA became stronger in the female reproductive tissues—ovary, style, and stigma (Figure 5A,B). In the male organs, only filaments were infected, while no signal was recorded within the anthers and in pollen grains (Figure 5C–F). DAPI staining implies that pollen grains before and after germination are viable (Figure 5E,F). Microscopic observations of generative and sperm cells during germination and tube growth further substantiated the lack of viruses in pollen viruses (Figure 5F). Ovuli fertilization is followed by seed development inside the ovary (Figure 5G). The green capsules originating from the mother carpels are heavily contaminated by viruses, while no fluorescent signal was recorded in the developing (Figure 5G) and maturing seed coat (Figure 5H).

Seed infection, including virus presence inside and/or on the seed coat, may result from either the maternal or paternal parent, or both [45]. Since all tested garlic pollen grains were free of viruses, the only means of embryo and endosperm virus infection are through maternal pathways. Maternal transmissions include several potential routes. Embryo invasion could result from infected meristematic tissue and derived megaspore mother cells; by direct invasion of the embryonic tissues via the suspensor as a transient pathway; or by infection of maternal seed parts, such as ovular integuments. For instance, in peas, the mosaic potyvirus infects the micropylar region at early seed development, moving via symplastic pore-like openings to access the suspensor cells and directly invade the embryo [46].

Studies on bulb onion [47] suggested that viruses are not transmitted by the true seed. We, therefore, anticipated similar results for garlic true seed, but the reality proved different. In our experiments, both the endosperm and embryo were infected by potyviruses (Figure 5I,J).

In many plant species, viruses can be transmitted by infected pollen both vertically and horizontally [48]. This is not the case for garlic potyviruses, as our results show that garlic anthers and pollen are free of these pathogens. Hence, they cannot contribute to viral infection of the developing seeds. In *Allium crystallinum, A. filidens*, and *A. fritschii*, which, like garlic, belong to the subgenus Allium, the epidermis of the mature anther consists of large bladder-shaped cells with strongly thickened outer walls covered by a toothed cuticle [49]. It is not clear yet whether in garlic virus transmission to pollen is blocked in the androecium tissue or the virus reproduction is eliminated during meiosis.

Another explanation for virus-free pollen in the infected plant can be a possible protection mechanism(s) during pollen differentiation. In general, the systemic viruses’ movement within the plants through vascular bundles or through plasmodesmata is protected by the capsid proteins. Since viruses are too large to move through unmodified plasmodesmata, they employ movement proteins dedicated to the enlarging of pores for the active transport of viral RNA [43,50,51]. Unlike our findings, Fabaceae, Asteraceae, and Poaceae potyviruses can be transmitted by pollen [52]. This process might be impaired by an unknown mechanism in garlic anthers. The lettuce mosaic potyvirus (LMV) was detected in the lettuce anthers, specifically in the tapetum, epidermis, endothecium, and the cytoplasm of the pollen grains [53]. The cowpea aphid-borne mosaic potyvirus (CABMV) was identified in pollens, anthers, and ovaries, but not in the seed coat [54].

Virus translocation and localization within the seed varies with plant species. For instance, in infected cucumber and melon plants, the fluorescence signal of Tobamovirus cucumber green mottle mosaic virus (CGMMV) was recorded in flowers, both before and following anthesis. Later, the virus was detected in the perisperm, endosperm envelope, which underlies the seed coat and surrounds the embryo [36]. Similarly, the soybean mosaic virus (SMV) is distributed in the seed testa, cotyledon, and radical tissue, but the seed embryo can remain free from SMV transmission [55]. In tomatoes, however, tomato brown rugose fruit virus (ToBRFV) is commonly spread only on the seed coat’s surface; hence, seed disinfection procedures effectively prevented seedlings from viral contamination [56,57]. In garlic, the seed coat was potyviruses-free; it consists of firm cells that probably provide a physical barrier for virus translocation (Figure 5H). These differences in sources of invasion, infection mechanisms, and virus localization in seeds draw attention to the most complicated plant–virus interaction and various mechanisms of virus transition via the reproductive system.

Plant viruses and other pathogens are transmitted both horizontally and vertically. Similar to other vegetatively propagated crops (e.g., potato) [58], garlic potyviruses can be transmitted within and between plant populations by aphids and thrips, or from one generation to another during clonal propagation. Here we provide the first evidence that garlic potyviruses can also be transmitted by true seeds produced from the infected mother plant.

Further research is required to assess the transmission of the other viral groups via the sexual reproductive system of garlic and elucidate the reason for the significant difference between various *Allium* species (e.g., bulb onion; see above). While Allexivirus and Carlaviruses are not reported as devastating pathogens, their interaction still can weaken garlic plants and reduce crop productivity [9].

Our observations suggest that the sexual progeny remains vigorous and highly productive for at least four or five years, despite the present findings on virus transmission via seed. Possibly, since seeds are free from other pathogens, such as fungi and some bacteria, sexually propagated plants are able to withstand viral infection.

In conclusion, garlic potyviruses are translocated from the infected mother plant during flower development or post-fertilization, while pollen remains virus-free and does not contribute to seed infection. Therefore, the main practical goal of virus-clean seed production requires careful maintenance of virus-free mother plants. Although garlic pollen is free of potyviral infection, the male parents’ plants also need to be protected from contamination, since viral infection weakens plants, reducing flowering ability and pollen production.

## Figures and Tables

**Table 1:** The specific primers for garlic viruses.

Virus	Forward Primer	Reverse Primer	Amplicon
Potyvirus LYSV	5′-GAG GAA AGT CAA TAC TTA AC-3′	5′-TGC TGT GTG CCT CTC CGT GTC CTC-3′	578 nt
Potyvirus OYDV	5′-GAG GAT GCA CAA TCA AG-3′	5′-TGC TGT GTG CCT CTC CGT GTC CTC-3′	714 nt
Allexiviruses GarV-A, B, C, D, E, X	5′-GAT CAC ATT CTG ATG CAT CCA CAC-3′	5′-CGT GAG GTC TTT GTT CAC GTC-3′	460 nt
Carlaviruses GLV, GCLV	5′-CTG AAT CAG ATT ATG AAG CTT TTG ATG C-3′	5′-CAA TCA CCC AGC TGG TAT TCG TC-3′	949 nt

**Table 2:** PCR analysis of garlic viruses in organs and tissues of clonally propagated fertile garlic genotypes, seeds, and seedlings.

Stage	Genotype	ALLEXI *	CARLA **	LYSV	OYDV
Foliage leaves	3376	+	-	+	+
	3379	+	-	-	+
Young inflorescences	3376	+	-	+	+
	3379	+	+	+	+
Mature flowers	3376	+	-	+	+
	3379	+	-	+	+
Seeds	87 (2015)	+	+	+	+
	87 (2016)	+	-	-	-
	87 (2017)	+	-	+	+
Seedlings	87 (2017)	+	-	+	+

* Including GarV-A, B, C, D, E, and X. ** Including GLV and GCLV.

## Data Availability

Not applicable.
